# Association between Micronutrient Levels and Chronic Spontaneous Urticaria

**DOI:** 10.1155/2015/926167

**Published:** 2015-10-22

**Authors:** Cheng-Han Wu, Efrem Eren, Michael Roger Ardern-Jones, Carina Venter

**Affiliations:** ^1^MSc Allergy, Faculty of Medicine, University of Southampton, Southampton SO16 6YD, UK; ^2^Dalin Tzu Chi Hospital, Buddhist Tzu Chi Medical Foundation, Chiayi County 62247, Taiwan; ^3^Southampton General Hospital, Southampton SO16 6YD, UK; ^4^The David Hide Asthma and Allergy Research Center, Isle of Wight PO30 5TG, UK; ^5^University of Portsmouth, Portsmouth PO1 2UP, UK

## Abstract

Previous reports have suggested a possible role for vitamin D in the etiology of chronic spontaneous urticaria (CSU); however, little information is available regarding the role of other micronutrients. We, therefore, analyzed vitamin D, vitamin B12, and ferritin levels in CSU patients (*n* = 282) from a preexisting database at Southampton General Hospital. Data were compared against mean micronutrient levels of the general population of the UK, obtained from the National Diet and Nutrition Survey. Vitamin D levels of CSU patients were found to be higher than those of the general UK population (*P* = 0.001). B12 levels were lower in patients with CSU (*P* < 0.001) than in the general population. Ferritin levels were found to be lower in male CSU patients than in the general male population (*P* = 0.009). This association between low B12 and iron levels and CSU might indicate a causal link, with micronutrient replacement as a potential therapeutic option.

## 1. Introduction

Vitamin D has been suggested to play a role in the aetiology of chronic spontaneous urticaria (CSU) [1–3]. However, the roles of other micronutrients in the development of CSU are unclear. In our clinical immunology practice, we routinely screen for micronutrient deficiencies in patients referred for allergy, immunology, and CSU. We observe that some of our CSU cases exhibit lessening of disease severity upon supplementing with low levels of vitamin D, vitamin B12, or iron, but this often happens alongside medical/dietary interventions that make it difficult to assess the true reason for symptom improvement. Lower vitamin D levels have previously been reported in CSU patients by Grzanka et al. and Thorp et al. [1, 2] However, the numbers of CSU patients enrolled in both of these studies were small (25 and 35, resp.); therefore, further studies are warranted. Furthermore, Mete et al. studied 33 patients with CSU and reported lower vitamin B12 levels in these patients compared to healthy controls [4]. Recently, Guarneri et al. reported significant improvement in urticaria after iron supplementation in a subgroup of CSU patients with iron deficiency and poor response to antihistamine. A higher prevalence of iron deficiency in CSU patients compared with healthy individuals was also reported in this study [5].

## 2. Materials and Methods

To determine micronutrient levels, vitamin D (25-hydroxyvitamin D), vitamin B12, ferritin, mean corpuscular volume (MCV), and mean corpuscular haemoglobin concentration (MCHC) levels of 1616 patients with CSU and other common presentations seen in the allergy clinic were extracted from a database at Southampton General Hospital (SGH). Patients between the ages of 19 and 64 years were included (mean age [years] ± SD, vitamin D: 41.2 ± 12.41 years; vitamin B12: 41.9 ± 12.40 years; ferritin: 41.0 ± 11.72 years). This was compared with the National Diet and Nutrition Survey (NDNS) conducted between 2008 and 2012 [6]. We also collected data on sex, date of birth, date of blood test, and the clinical descriptions provided when testing was requested. For vitamin D analysis, we investigated a number of samples collected during different months to determine the potential influence of seasonal variation.

According to the clinical descriptions, only nutrient investigations requested for CSU were included. Patients known to have received nutrient replacement were excluded. To minimize bias resulting from supplementation of nutrients, we excluded data from repetitive tests, unless the nutrient levels remained low or the first result was so high that further supplementation was very unlikely. Testing requested for hereditary angioedema, angioedema related to angiotensin-converting enzyme inhibitors, physical urticaria, acute urticaria/angioedema, urticarial vasculitis, or autoinflammatory syndrome was excluded. Also patients with diagnoses of celiac disease or upper gastrointestinal tract surgery were excluded.

We analysed mean nutrient levels and the proportion of CSU patients with a nutrient deficiency. Data from NDNS were age-matched to our cohort to establish normal population micronutrient levels [6]. Differences between our data and mean nutrient concentrations in the NDNS sample were analysed by using *t*-tests. All statistical analyses were performed using GraphPad Prism software, version 6 (La Jolla, CA, USA). Statistical significance was defined at *P* values of 0.05 or less.

## 3. Results and Discussion

Of the 1616 patients extracted from the SGH database, 282 patients were identified as having CSU. Most of the CSU patients were female. Vitamin D levels were higher in the CSU patients than in the general UK population (mean vitamin D ± SD, 51.4 ± 27.03 versus 45.4 ± 24.84 nmol/L; *P* = 0.001; Table 1). However, the majority of people with urticaria (54.6%) had vitamin D levels below 50 nmol/L. The proportion of samples collected in summer and autumn (between June and November) was 54.1%.

Compared with the general UK population, CSU patients had significantly lower levels of vitamin B12 but were still within the normal range (mean vitamin B12 ± SD, 284 ± 108.4 versus 359 ± 131.2 ng/L; *P* < 0.001). In addition, lower ferritin levels were observed in CSU patients of both sexes (mean ferritin ± SD, female: 48 ± 44.4 versus 56 ± 53.6 ug/L; male: 101 ± 70.3 versus 140 ± 100.6 ug/L), but the difference was not significantly lower in female CSU patients (female: *P* = 0.096, versus male: *P* = 0.009) compared to the general female population. The proportions of patients with low B12 (below 130 ng/L) and low ferritin (below 11 ug/L for females and below 24 ug/L for males) levels were 4.5% and 8.5%, respectively. Because full blood count (FBC) testing is recommended by most mainstream urticaria guidelines, we investigated the usefulness of MCV in screening for B12 or iron deficiencies. Out Of 16 patients with low ferritin, only six had MCV values below 80 fL and none had low MCHC. On the other hand, none of the eight patients with low vitamin B12 levels exhibited MCV values above 100 fL.

Therefore, in contrast with the results of previous studies [1, 2], we observed higher vitamin D levels in our CSU population in comparison with the UK general population. One possible explanation might be the reported effect of latitude in the wide variation of vitamin D levels in the UK population and the southern location of our catchment area [7]. However, we cannot prove it due to the lack of data on vitamin D levels of the general population in Southampton.

Although the previous cohort reported by Mete et al. (*n* = 33) [4] was much smaller than that in our current study, both studies suggest that lower B12 levels are associated with CSU. We also observed an association between iron and CSU, in agreement with a previous study [5]. However, the prevalence of iron deficiency in CSU patients was much lower in our study (16/188 versus 81/122). Even though the low sensitivity of erythropoietin parameters in the diagnosis of B12 or iron deficiency has been previously reported [8, 9], it is worth noting that FBC alone is not helpful in excluding B12 or iron deficiencies.

We acknowledge the limitations of this study, including the fact that we did not record dietary or medication history for our cohort; it is possible that variances in diet account for micronutrient differences. For example, an increasing number of patients are taking vitamin D supplements due to increased public awareness. Nevertheless, we suggest that the lower vitamin B12 and iron levels in CSU patients in our study may indicate that these micronutrients are interesting possible candidates for a role in the initiation or possibly maintenance of CSU. Although the mechanism is still unknown, it could be hypothesized that micronutrients regulate cytokine release following mast cell degranulation by their immunomodulatory properties. Wheatley reported an inverse relationship between vitamin B12 and tumour necrosis factor alpha [10], which is upregulated in CSU patients [11]. Iron-induced decreases in the number of mast cell granules and percentage of mast cell degranulation have also been observed in vitro [12].

## 4. Conclusions

Whether the “low-normal” B12 and ferritin levels seen in most of our CSU cohort reflect either inadequate diets or an etiological role is unclear; however the effect of more general supplementation remains to be established, and a dietary intervention study would be an exciting prospect in this disease area. Further information regarding dietary intake and food avoidance habits of patients with CSU will serve to build on our observations.

## Figures and Tables

**Table 1:** Differences in micronutrient levels between SGH patients and the general UK population, aged 19 to 64 years.

	CSU patients in SGH (mean ± SD)	Normal population in NDNS (mean ± SD)	*P* value
Vitamin D levels (nmol/L)	51.4 ± 27.03 (*n* = 225)	45.4 ± 24.84 (*n* = 1321)	**0.001**
Vitamin D levels (nmol/L), females	53.1 ± 27.51 (*n* = 163)	47.3 ± 25.61 (*n* = 770)	0.010
Vitamin D levels (nmol/L), males	46.8 ± 25.38 (*n* = 62)	43.5 ± 23.87 (*n* = 551)	0.306
Vitamin B12 levels (ng/L)	284 ± 108.4 (*n* = 176)	359 ± 131.2 (*n* = 1320)	**<0.001**
Vitamin B12 levels (ng/L), females	281 ± 113.8 (*n* = 133)	359 ± 132.2 (*n* = 768)	<0.001
Vitamin B12 levels (ng/L), males	295 ± 89.7 (*n* = 43)	359 ± 130.2 (*n* = 552)	0.002
Ferritin levels (ug/L)	61 ± 57.0 (*n* = 188)	97 ± 90.2 (*n* = 1329)	**<0.001**
Ferritin levels (ug/L), females	48 ± 44.4 (*n* = 140)	56 ± 53.6 (*n* = 775)	0.096
Ferritin levels (ug/L), males	101 ± 70.3 (*n* = 48)	140 ± 100.6 (*n* = 554)	0.009

Definition of micronutrient deficiency: vitamin D < 50 nmol/L; vitamin B12 < 130 ng/L; ferritin < 11 ug/L for females; ferritin < 24 ug/L for males. NDNS: the National Diet and Nutrition Survey; SGH: Southampton General Hospital.
